# Supplementary material for: Unexpected Method of High-Viscosity Shear Thickening Fluids Based on Polypropylene Glycols Development via Thermal Treatment
Source: Materials (Basel). 2022 Aug 24;15(17):5818. doi: 10.3390/ma15175818 (PMC9457279; doi:10.3390/ma15175818)
Supplement: Supplementary file 1 [file materials-15-05818-s001.zip › materials-1831065-SI.pdf]

materials

MDPI

Supplementary Materials

For

Unexpected Method of High Viscosity Shear Thickening Fluids Based on Poly-propylene Glycols Development *via* Thermal Treatment

Mariusz Tryznowski [1,*], Tomasz Gołofit [2], Selim Gürgen [3], Patrycja Kręcisz [4] and Marcin Chmielewski [5,6]

[1] Faculty of Mechanical and Industrial Engineering, Warsaw University of Technology, Narbutta 85, 02-524 Warsaw, Poland
[2] Faculty of Chemistry, Warsaw University of Technology, Noakowskiego 3, 00-664 Warsaw, Poland
[3] Department of Aeronautical Engineering, Eskişehir Osmangazi University, 26040 Eskişehir, Turkey
[4] Faculty of Material Engineering, Warsaw University of Technology, Wołoska 141, 02-507 Warsaw, Poland
[5] Institute of Microelectronics and Photonics, Łukasiewicz Research Network, Lotników 32/46, 02-668 Warsaw, Poland
[6] National Centre for Nuclear Research, Materials Research Lab, Świerk, 05-400 Otwock, Poland
* Correspondence: mariusz.tryznowski@pw.edu.pl

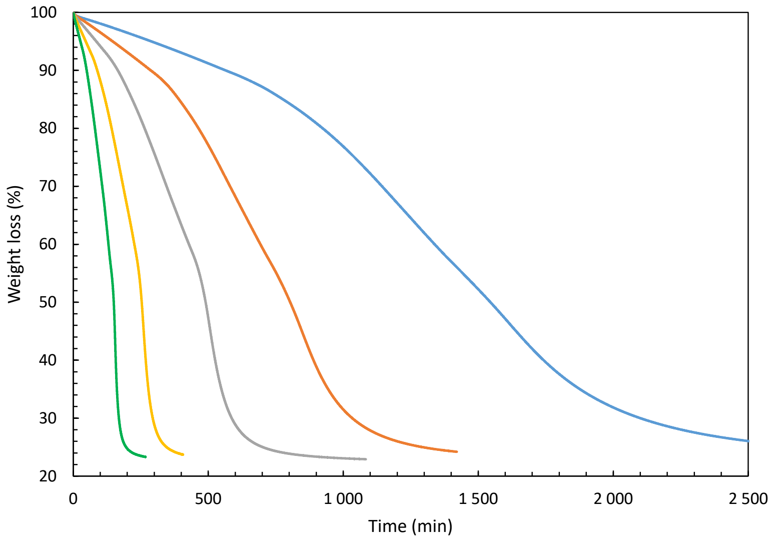

Figure S1. TG curves for STF100-24 at constant temperatures: 100°C (blue line), 110°C (orange line), 120°C (grey line), 130°C (yellow line) and 140°C (green line)

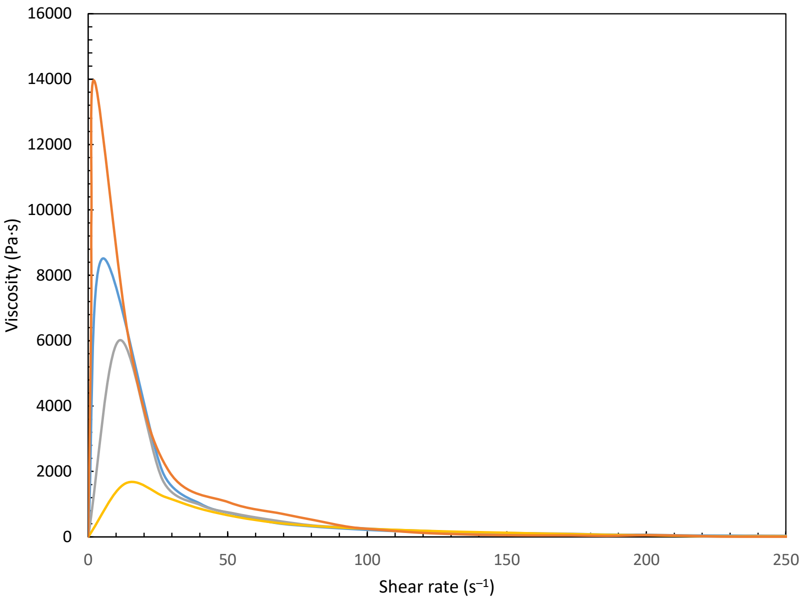

Figure S2. Viscosity vs. shear rate for STF1000-24 after thermal treatment: yellow line – STF1000-24 prior thermal treatment; gray line – approx. 15% weigh loss of STF1000-24; blue line – approx. 20% weigh loss of STF1000-24; orange line – approx. 25% weigh loss of STF1000-24.
